# Comparative effects of virtual reality training and sensory motor training on bone morphogenic proteins and inflammatory biomarkers in post-traumatic osteoarthritis

**DOI:** 10.1038/s41598-020-72587-2

**Published:** 2020-09-28

**Authors:** Gopal Nambi, Walid Kamal Abdelbasset, Shereen H. Elsayed, Mona A. Khalil, Saud M. Alrawaili, Saud F. Alsubaie

**Affiliations:** 1 Department of Health and Rehabilitation Sciences, College of Applied Medical Sciences, Prince Sattam Bin Abdulaziz University, Al-Kharj, Saudi Arabia; 2 Department of Physical Therapy, Kasr Al-Aini Hospital, Cairo University, Giza, Egypt; 3 Department of Rehabilitation Sciences, Faculty of Health and Rehabilitation Sciences, Princess Nourah Bint Abdulrahman University, Riyadh, Saudi Arabia; 4 Department of Biochemistry, Faculty of Medicine for Girls, Al-Azhar University, Cairo, Egypt

**Keywords:** Biochemistry, Biological techniques, Biomarkers, Health care, Medical research, Rheumatology

## Abstract

The objective of this study is to compare the effects of virtual reality training (VRT) and sensory-motor training (SMT) in bone morphogenetic proteins (BMP) and inflammatory biomarkers expression in post-traumatic osteoarthritis (PTOA) after the anterior cruciate ligament injury. Through a simple random sampling method, 60 eligible participants were allocated into VRT (n = 20), SMT (n = 20), and control groups (n = 20). They underwent training programs for 4 weeks. Clinical (pain intensity and functional disability) and biochemical (bone morphogenic proteins and inflammatory biomarkers) values were measured at baseline, after 4 weeks, 8 weeks and 3 months follow up. Four weeks following training, the VRT group shows more significant changes in pain intensity and functional disability than SMT and control groups (P < 0.001). Bone morphogenic protein (BMP) measures such as BMP 2, 4, 6, and 7 don’t show any significant changes between the groups. But at the same time, the VRT group shows positive improvement in inflammatory biomarkers (CRP, TNF-α, IL-2, IL-4, IL-6) analysis than the other two groups (P < 0.001). Our study suggests that including virtual reality training in PTOA shows beneficial changes in pain, functional disability, and modification of inflammatory biomarkers than sensory-motor training, but at the same time it shows a negligible effect on bone morphogenic proteins.

## Introduction

Knee osteoarthritis (OA) is a common degenerative condition occurring mainly due to some occupational injuries or due to sports activities. Post-traumatic osteoarthritis (PTOA) is a type of OA; in which the occur due to abnormal joint mechanisms after any soft tissue injuries. These abnormal joint mechanisms put excessive or uneven pressure on the joint surfaces and lead to cartilage degeneration^1^. Anterior cruciate ligament (ACL) injury is one of these soft tissue injuries leading to this consequence^2^. It is noted that amateur football players are more prone to ACL injury due to their rotatory movements at the knee during the game. Particularly, in these players marked imbalance between hamstring and quadriceps muscle strength is also identified^3^. PTOA occurrence due to ACL injury is about 18.8% and it is remarkably higher than that observed in other field games^4^. The relative causes for ACL injuries in football are; altered Hamstring/Quadriceps (H/Q) ratio, reduced lower limb muscle strength, and any other deformities in the lower extremity. Overall, these factors directly influence the players’ attitude and performances during the game^5^.

Recent reports show that ACL injury has a high probability of developing PTOA irrespective of its treatment^6^. It slowly affects all parts of the joint such as; joint capsule, synovial membrane, synovial fluid, articular cartilage, and subchondral bone. Studies report that poor physical training and maintaining bad posture during the game are the main causes of this problem^7^. It puts unnecessary psychological stress on the players and activates the medical team to understand the cause of the mechanism of PTOA after ACL injury. It is found that arthrogenic muscle inhibition (AMI) in the quadriceps muscle following ACL injury is a major cause for knee PTOA^8^. Apart from this, uneven weight distribution, reduced shock absorption, and wrong biomechanical forces lead to this problem^9^. It is found that subtle ACL rupture may injure the cartilage and trigger the metabolic (Bone morphogenic protein—BMP) and inflammatory changes in the joint and bone. These changes generally protect and treat the cartilage from degeneration occurring during osteoarthritis. BMP has a strong anabolic effect on chondrocytes by activating the synthesis of cartilage matrix components. It also controls and activates the proteoglycan and collagen synthesis and has a positive role in inflammatory cytokines^10^. The common inflammatory changes like cellular infiltration, cytokine production, stimulation of chondrocytes, and synoviocytes can also be seen in PTOA^11^.

Surgical and non-surgical approaches have shown a positive improvements in PTOA following ACL injury. However, the neuromuscular control and muscle properties over the joint are not obtained properly and the H/Q imbalance also persists for a long time after injury^12^. Recent studies observed that integrated rehabilitation training programs improve neuromuscular control, muscular symmetry, cartilage morphology and lower the levels of inflammatory reactions in patients with PTOA following ACL injury^13^. Virtual reality training (VRT) is the latest technology in the field of sport rehabilitation, which works on the principle of making virtual environments through special software on the computer. It has the ability to facilitate the visual and auditory cues and in turn activate the neuromuscular system^14^. This sensory feedback stimulates the higher centers in the brain and stimulates the pain depression pathway, which ameliorates the pain sensation in the joint^15^. It is suitable for pain rehabilitation programs because it offers work to the patient within their pain limits. This enhances neuromuscular control and improves muscle properties^16^. The real-time virtual environments are set as per the requirement of the therapist and the patient, which have an important role in the treatment outcomes^17,18^. Studies report that VRT improves the pain intensity, functional status, and blood serum levels of stress hormones^19,20^. Nevertheless, so far no studies have been conducted to find the effects of VRT on bone morphogenic proteins such as BMP 2, 4, 6, and 7 and inflammatory biomarkers (C reactive protein—CRP, Tumor necrosis factor—TNF-α, Interleukins (IL-2, IL-4, IL-6) expression levels in PTOA after ACL injury.

Furthermore, it is the high time to find the effects and mechanism of BMP and inflammatory biomarkers in PTOA following ACL injury. Obtaining knowledge about these training programs brings the treatment program for PTOA in a favorable way. Moreover, these training programs would modify the risks and the possible consequences of ACL injury. Hence, the aim of this study was to find and compare the effects of virtual reality training and sensory-motor training on bone morphogenetic proteins and inflammatory biomarkers expression in post-traumatic osteoarthritis after the ACL injury. Also, the reports of this study would be helpful for the physical therapists to select a proper rehabilitation program and for the players to reduce the risk of PTOA and improve the prognosis.

## Materials and methods

### Trial design

This trial was a randomized, double-blinded, parallel-group controlled study and the participants were randomized through a computer-generated random table. They were allocated equally into three groups by using the group information in the sealed envelopes. Totally sixty (N = 60) participants were selected and allocated to the three groups namely: virtual reality training VRT; n = 20, Sensory-motor training SMT; n = 20 and Control; n = 20 groups. The trial was accredited by department ethical committee (DEC), Department of physical therapy and health rehabilitation, Prince Sattam Bin Abdulaziz University, Al-Kharj, Saudi Arabia. The trial was performed as per the guidelines of the declaration of Helsinki 1964 and submitted in the format of consolidated standards of reporting trials (CONSORT) guidelines with reference no RHPT/020/004.

The trial was performed in the Department of Physical Therapy and Health Rehabilitation, Prince Sattam Bin Abdul Aziz University, Al-Kharj, Saudi Arabia. Participants were recruited from the University Hospital and King Khalid hospital, Al-Kharj, Saudi Arabia. The orthopedic surgeon at the outpatient clinic evaluated the participants for participating in the study as per the selection criteria.

### Participants

At first, all the selected participants were informed about the study procedures, its merits, and demerits through a study booklet. The participants who agreed and signed the informed consent were included in the final selection. The inclusion criteria were; male football players, age 18–25 years, chronic (≥ 3 months) PTOA following ACL injury, as diagnosed by an orthopedic surgeon (through physical examination and radiological findings), and pain rating 4–8 in visual analog scale (VAS) were permitted. Participants with other orthopedic, neural, systemic, psychological, and awaiting for surgery were excluded. Participants who underwent any other treatment and physical training were also excluded.

### Interventions

**Figure 1:**
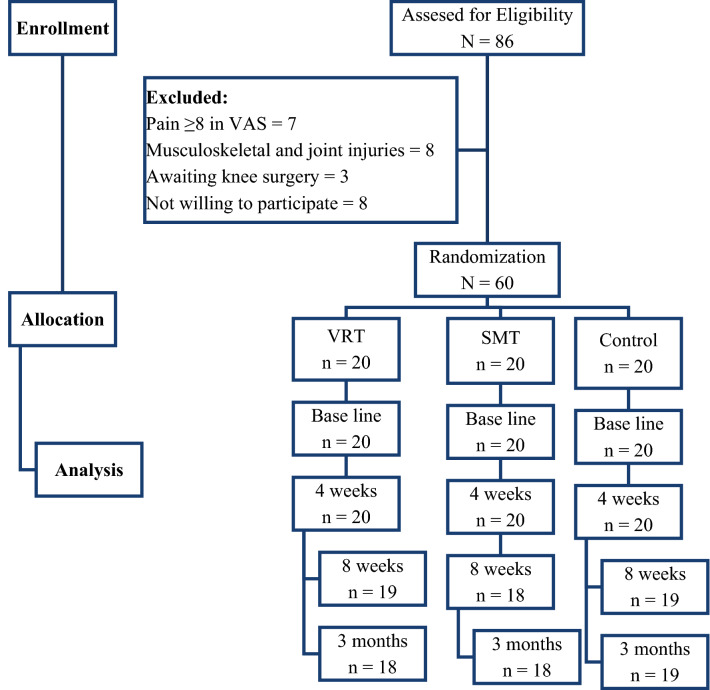
Flow chart showing the study details.

After final selection, each group had 20 participants and they underwent four weeks rehabilitation program as per the guidelines of the ethical committee. The respective rehabilitation programs were carried out by a minimum of five years’ experience as physiotherapists. During the selection procedure, we have excluded seven participants with more pain (≥ 8 in VAS scale), eight participants with other orthopedic injuries, three with awaiting surgery and eight were excluded due to not willing to participate in the trial (Fig. 1).

The participants in the VRT group received training with the device (Pro-Kin system PK 252 N Techno body, Italy) focused on the improvement of knee muscles. Personal training for all the participants in the device was given to obtain knowledge about the usage of this training and also got an idea about VRT. The participant was asked to stand by keeping the affected limb in the virtual platform and informed to watch the computer display screen. The task selected in this trial was shooting the balls, in which the task was operated and managed by moving the knee joint forward, backward, left, and right as per the requirements. The participant was permitted to do all the movements of the knee within his efficiency and pain limits. The progression of the exercise can be done by increasing the difficulty level which offers more muscle activity and joint movement. It was done by increasing the number of balls, change of throwing angles, increase the rate of the shoot, increase the rate of displaying of balls, and the number of balls appearing around the participant. This training was continued for 20 min session for two sessions for 5 days in a week for 4 weeks^21^.

The participants in the SMT group performed sensorimotor training exercises in three stages and the exercises were prescribed in a graded manner, where the participant performed and completed all the stages. After completing the first level, they were allowed to the second and third levels. In the initial phase (static) the participant was informed to stand straight for 30 s on a hard plate and 30 s on a foam plate. Next, the participant was instructed to stand on a single leg (affected side) with closed eyes for 20 s on a hard plate and 20 s on a foam plate, followed by a semi knee bending position for 10 s. In the next phase (Dynamic), the participant was informed to perform forward kicking for 30 s and T-band kicking for 30 s. Finally, in the last phase (functional) the participant was informed to do toe jumping for 20 m and heel jumping for 20 m. At last, the participant was asked to perform bilateral and unilateral squatting exercises for 10 repetitions with and without the support of a wall. The exercises were continued for 5 repetitions in one set for 3 sets with 3 min rest between the sets for 5 days in a week for 4 weeks^22^.

In the control group, the participants underwent supervised conventional exercise programs for the knee muscles. At first, the supervisor demonstrated the exercises to the participant and asked them to repeat the exercises under his supervision and the mistakes were clarified. These exercises laid special stress on the quadriceps, hamstrings, glutei, and calf muscles. The participants performed 10–15 repetitions in one set for 3 sets with 1-min rest between the sets for 5 days in a week for 4 weeks. Stretching focused on each muscle group for 3 repetitions for 15 s per muscle group^23^.

### Outcome variables

#### Pain intensity

The intensity of pain at rest was measured by a visual analog scale (VAS). It is a 10 cm long horizontal line denoting starting point with “no pain” and the endpoint with “maximum intolerable pain”. Participants were asked to mark their level of pain perception in the last 24 h and the distance was measured from the starting point and noted. It is a reliable and valid tool to measure pain intensity in orthopedic conditions^24^.

#### Functional disability

The functional ability of the participant was evaluated with the Arabic version of the Western Ontario and McMaster Universities Arthritis Index (WOMAC) scale. It has a total of 24 questions categorized into 3 parts; pain, stiffness, and physical function. Each participant answered the questions on a 0–4 Likert scale (0—none, 1—mild, 2—moderate, 3—severe, and 4—extreme). The minimum score indicates “no disability” and the maximum score indicates “severe disability”. It is a reliable and valid tool to measure Knee disability^25^.

#### Bone morphogenic proteins

The fasting 10 ml serum blood sample was collected at morning 8 O'clock by a lab attendant. The sample was processed in a standard procedure, in which after centrifugation the serum plasma sample was stored at − 70 °C. BMP ELISA kit (Elab science, Co Ltd, China), ELISA kit was used to analyze the bone morphogenic proteins; BMP 2, 4, 6, and 7 in the serum plasma concentrations. The kit was used as per the instructions of the company. The procedure was done at different intervals^10^.

#### Inflammatory biomarker

The fasting 10 ml serum blood sample was collected at morning 8 O'clock by a lab attendant. Through the centrifugation procedure, the serum was separated and kept at − 700 °C in the freezer. Serum levels of inflammatory cytokines such as CRP, TNF-α, IL-2, IL-4, and IL-6 were evaluated by enzyme-linked immunosorbent assay (My Biosource, Multiplex human cytokine ELISA kit, CA, USA) procedure. The kit was used as per the instructions of the company. The minimum and maximum values were noted for each biomarker and the average was considered for data analysis.

### Sample size

The number of samples required for this study was calculated by data from a previous study, in which the number of samples required to detect a standard mean difference of (SMD = 40%) with standard deviation (SD = 0.5) in pain intensity was 18 in each group. It was obtained by considering the power (1 − β = 80) with significance level α = 0.05. When considering 20% dropout the total subjects required for each group became 2012.

### Blinding

In spite of this study design, it was not feasible to mask the physiotherapist who was providing treatment to the participants, but at the same time, participants and the evaluating therapist were blinded. All the participants were informed not to share their treatment procedures with the fellow participants and the evaluating therapist. Therefore, the treating and evaluating therapists were different persons, and the outcomes were measured at baseline, after 4 weeks, 8 weeks and 3 months follow up.

### Statistical analysis

Participant’s baseline demographic and clinical characteristics were evaluated to decide the study homogeneity by using the Kolmogorov–Smirnov test. Outcome data were presented as mean and standard deviation and 3 × 4 (Group × Time) multiple analysis of variance (MANOVA) was performed to know the group and time effect. A repeated measure of ANOVA was performed to determine significant differences within the groups. Oneway ANOVA test was used for comparison between the groups and the statistical significance level was set at P < 0.05. SPSS software (version 20.0) SPSS Inc, Chicago, Illinois, USA was used for all statistical analyses.

## Results

### Participants

Among eighty-six participants referred from local hospitals, N = 60 were eligible to participate in the study and allocated (n = 20) each to VRT, SMT, and control groups. However, two participants each from VRT and SMT group and one participant from the control group were dropped out during the study due to personal inconveniences. Hence intention to treat analysis principle was presumed in this study for the data analysis. The baseline demographic characters like age, height, weight, and Body Mass Index (BMI) were analyzed and showed no significant difference (P > 0.05), which indicates a homogenous population. Furthermore, the clinical characters like Oxygen volume (VO2), years of playing and duration of pain were also analyzed to find the fitness to participate in the treatment protocol and these clinical characters were also not shown any statistical difference (P > 0.05) between the three groups (Table 1).

**Table 1:** One way ANOVA analysis of demographic details of VRT, SMT and Control group.

Sr. no	Variable	VRT Mean and SD	SMT Mean and SD	Control Mean and SD	P-value
1	Age (y)	22.8 ± 1.3	22.6 ± 1.4	21.9 ± 1.3	0.090
2	Height (m)	1.74 ± 0.16	1.75 ± 0.15	1.73 ± 0.16	0.921
3	Weight (kg)	65.5 ± 3.1	66.7 ± 2.9	67.6 ± 2.8	0.085
4	BMI (kg/m^2^)	22.4 ± 1.6	22.5 ± 1.5	23.1 ± 1.4	0.288
5	VO_2_peak (ml/kg/min)	37.5 ± 3.6	37.3 ± 3.9	38.2 ± 4.1	0.743
6	HR (beats/min)	168 ± 7.2	169 ± 7.1	166 ± 7.5	0.419
7	Years of playing (y)	4.9 ± 2.1	5.2 ± 1.9	5.4 ± 1.8	0.715
8	Duration of pain (m)	4.8 ± 1.2	4.2 ± 1.1	5.1 ± 1.3	0.630

*VRT* virtual reality training, *SMT* sensory motor training, *SD* standard deviation, *BMI* Body Mass Index, *VO*_*2*_ oxygen volume, *HR* heart rate, *y* years, *m* months.

### Pain and functional disability

**Figure 2:**
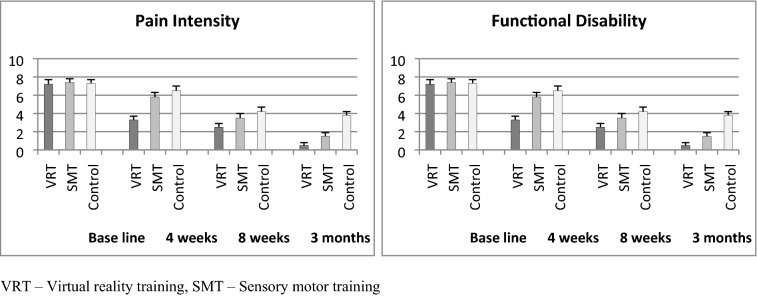
Mean values of VAS and WOMAC scores in VRT, SMT and control group.

The baseline values of pain intensity by VAS and functional disability by WOMAC did not show any statistical difference (P > 0.05) between the three groups, which indicates the normal distribution of samples. The 3 × 4 (Group × Time) multiple analysis of variance (MANOVA) at baseline, 4th week, 8th week and 3 months follow up showed significance difference (P < 0.001) in VAS and WOMAC scores between the groups. The intergroup analysis by one way ANOVA also showed significant differences (P < 0.001) in both variables at various intervals (Table 2). The post hoc Bonferroni correction analysis showed more probability of changes in the VRT group than SMT and control groups. The Table 2 and Fig. 2 shows more percentage of improvement in pain and functional disability in the VRT group than SMT and control groups.

**Table 2:** Pre and post VAS and WOMAC analysis of VRT, SMT and Control group.

Sr. no	Variable		VRT Mean and SD	SMT Mean and SD	Control Mean and SD	P-value
1	Pain intensity (VAS)	Base line	7.2 ± 0.5	7.4 ± 0.4	7.3 ± 0.4	0.355
		4 weeks	3.3 ± 0.4	5.8 ± 0.5	6.5 ± 0.5	0.001*
		8 weeks	2.5 ± 0.4	3.5 ± 0.5	4.2 ± 0.5	0.001*
		3 months	0.5 ± 0.3	1.5 ± 0.4	3.8 ± 0.4	0.001*
		P-value	0.001*	0.001*	0.001*	
2	Functional disability (WOMAC)	Base line	72.33 ± 4.2	72.47 ± 4.5	71.22 ± 3.8	0.587
		4 weeks	34.11 ± 3.4	56.32 ± 3.8	62.28 ± 3.2	0.001*
		8 weeks	22.41 ± 2.1	32.35 ± 2.8	52.28 ± 3.2	0.001*
		3 months	11.21 ± 2.1	25.32 ± 2.1	35.22 ± 2.8	0.001*
		P-value	0.001*	0.001*	0.001*	

*Significant, *VRT* virtual reality training, *SMT* sensory motor training, *SD* standard deviation.

### Bone morphogenic proteins

**Figure 3:**
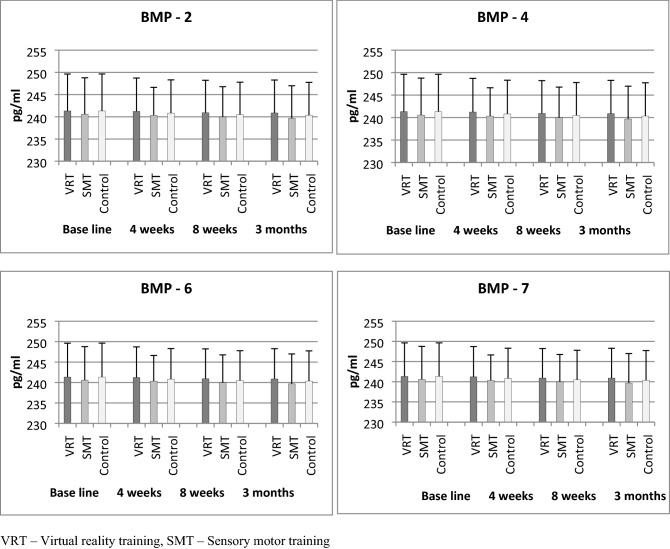
Mean values of BMP-2, BMP-4, BMP-6 and BMP-7 levels in VRT, SMT and control groups.

The baseline values of BMP 2, 4, 6, and 7 did not show any statistical difference (P > 0.05) between the three groups, which indicates the normal distribution of samples. The 3 × 4 (Group × Time) multiple analysis of variance (MANOVA) at baseline, 4th week, 8th week, and 3 months follow up did not show any statistical difference (P > 0.001) in BMP 2, 4, 6, and 7 scores between the groups. The intergroup analysis by one way ANOVA also did not show any significant differences (P > 0.001) in all the BMP variables at various intervals (Table 3). The post hoc Bonferroni correction analysis showed an equal probability of changes in VRT, SMT, and control groups. The Table 3 and Fig. 3 shows the little or negligible amount of improvement in bone morphogenic proteins in the VRT group than SMT and control groups.

**Table 3:** Pre and post bone morphogenic protein analysis of VRT, SMT and control group.

Sr. no	Variable		VRT Mean and SD	SMT Mean and SD	Control Mean and SD	P-value
1	BMP-2 (pg/ml)	Base line	657.12 ± 8.1	656.33 ± 8.9	656.22 ± 8.5	0.935
		4 weeks	656.45 ± 7.1	656.22 ± 7.2	655.72 ± 7.1	0.946
		8 weeks	656.45 ± 7.2	656.01 ± 7.3	655.48 ± 9.2	0.928
		3 months	655.92 ± 7.5	656.23 ± 7.4	655.28 ± 9.8	0.934
		P-value	0.967	0.999	0.987	
2	BMP-4 (pg/ml)	Base line	643.21 ± 7.9	643.66 ± 7.8	642.89 ± 7.6	0.951
		4 weeks	642.88 ± 7.2	642.58 ± 7.6	642.28 ± 7.4	0.967
		8 weeks	642.43 ± 7.3	642.45 ± 7.2	642.15 ± 7.4	0.989
		3 months	642.15 ± 7.6	642.11 ± 7.5	642.03 ± 7.9	0.984
		P-value	0.971	0.923	0.841	
3	BMP-6 (pg/ml)	Base line	698.88 ± 9.2	699.78 ± 9.4	698.76 ± 9.5	0.931
		4 weeks	698.57 ± 8.5	699.46 ± 8.3	698.59 ± 8.5	0.930
		8 weeks	698.36 ± 8.2	699.35 ± 8.3	698.28 ± 8.3	0.901
		3 months	698.12 ± 8.1	699.12 ± 8.2	698.11 ± 8.3	0.904
		P-value	0.993	0.994	0.995	
4	BMP-7 (pg/ml)	Base line	241.32 ± 8.3	240.57 ± 8.2	241.33 ± 8.3	0.946
		4 weeks	241.22 ± 7.5	240.33 ± 6.3	240.81 ± 7.5	0.924
		8 weeks	240.92 ± 7.3	239.97 ± 6.8	240.49 ± 7.3	0.915
		3 months	240.88 ± 7.4	239.68 ± 7.3	240.34 ± 7.4	0.977
		P-value	0.997	0.980	0.930	

*VRT* virtual reality training, *SMT* sensory motor training, *BMP* bone morphogenic proteins, *SD* standard deviation.

### Inflammatory biomarker

**Figure 4:**
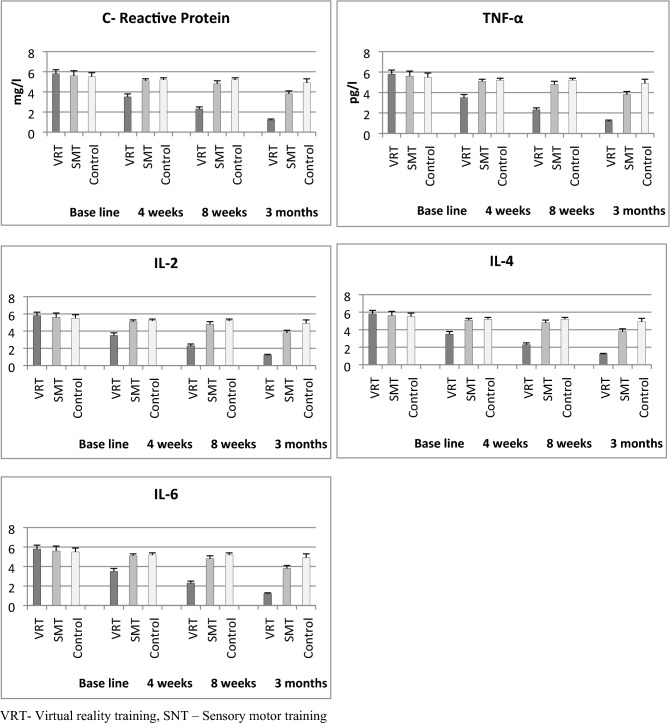
Mean values of CRP, TNF-α, IL-2, IL-4 and IL-6 levels in VRT, SMT and control group.

The baseline values of CRP, TNF-α, IL-2, IL-4, and IL-6 did not show any statistical difference (P > 0.05) between the three groups, which indicates the normal distribution of samples. The 3 × 4 (Group × Time) multiple analysis of variance (MANOVA) at baseline, 4th week, 8th week and 3 months follow up show significance difference (P < 0.001) in CRP, TNF-α, IL-2, IL-4, and IL-6 scores between the groups. The intergroup analysis by one way ANOVA also shows significant differences (P < 0.001) in all inflammatory biomarker variables at various intervals (Table 4). The post hoc Bonferroni correction analysis shows more probability of changes in the VRT group than SMT and control groups. The Table 4 and Fig. 4 shows more percentage of improvement in all inflammatory biomarker variables in the VRT group than SMT and control groups.

**Table 4:** Pre and post pro inflammatory biomarker analysis of VRT, SMT and Control group.

Sr. no.	Variable		VRT Mean and SD	SMT Mean and SD	Control Mean and SD	P-value
1	CRP (mg/l)	Pre	1.55 ± 0.4	1.54 ± 0.4	1.52 ± 0.4	0.971
		4 weeks	1.08 ± 0.4	1.48 ± 0.3	1.45 ± 0.2	0.001*
		8 weeks	0.57 ± 0.2	1.23 ± 0.3	1.38 ± 0.3	0.002*
		3 months	0.32 ± 0.2	0.98 ± 0.4	1.29 ± 0.4	0.001*
		P-value	0.001*	0.001*	0.173*	
2	TNF-α (Pg/ml)	Pre	15.92 ± 0.7	15.48 ± 0.6	15.92 ± 0.8	0.832
		4 weeks	10.47 ± 0.4	14.52 ± 0.4	15.32 ± 0.4	0.001*
		8 weeks	8.23 ± 0.5	13.21 ± 0.4	14.56 ± 0.5	0.001*
		3 months	7.21 ± 0.3	11.89 ± 0.4	14.11 ± 0.4	0.001*
		P-value	0.001*	0.001*	0.001*	
3	IL-2	Pre	12.89 ± 1.1	12.26 ± 0.9	12.92 ± 1.2	0.990
		4 weeks	13.56 ± 0.7	12.88 ± 0.7	12.98 ± 0.9	0.015*
		8 weeks	14.98 ± 0.6	13.74 ± 0.6	13.21 ± 0.7	0.001*
		3 months	16.02 ± 0.6	13.93 ± 0.6	13.38 ± 0.5	0.001*
		P-value	0.001*	0.001*	0.313	
4	IL-4	Pre	39.94 ± 0.7	39.43 ± 0.8	38.95 ± 0.8	0.057
		4 weeks	48.65 ± 0.8	43.61 ± 0.7	39.89 ± 0.7	0.001*
		8 weeks	59.32 ± 0.7	52.38 ± 0.8	41.32 ± 0.7	0.001*
		3 months	65.11 ± 0.9	56.38 ± 0.9	42.28 ± 0.8	0.001*
		P-value	0.001*	0.001*	0.075	
5	IL-6	Pre	5.8 ± 0.4	5.6 ± 0.5	5.5 ± 0.4	0.094
		4 weeks	3.5 ± 0.3	5.1 ± 0.2	5.2 ± 0.2	0.001*
		8 weeks	2.3 ± 0.2	4.8 ± 0.3	5.2 ± 0.2	0.001*
		3 months	1.2 ± 0.1	3.8 ± 0.3	4.9 ± 0.4	0.001*
		P-value	0.001*	0.001*	0.603	

*Significant, *VRT* virtual reality training, *SMT* sensory motor training, *CRP* C reactive protein, *TNF* tumor necrosis factor, *IL* interleukin, *SD* standard deviation.

## Discussion

This randomized controlled study was done to investigate the clinical, bone morphogenic, and inflammatory effects of different training protocols in PTOA after ACL injury. Virtual reality training, sensory-motor training, and conventional knee training protocols had been carried out for the three groups respectively for 4 weeks and the primary and secondary outcome measures were measured at different intervals. Reports suggest that the primary variables such as pain and functional disability improved positively in the VRT group than the other two groups. At the same time, the secondary variable bone morphogenic proteins (BMP 2, 4, 6, and 7) did not show any statistical changes between the three groups. The other variable, inflammatory biomarkers (CRP, TNF-α, IL-2, IL-4, and IL-6) showed significant differences between the groups, and post hoc analysis showed the VRT group had more positive changes than SMT and control groups.

This study also showed clinical improvement in pain intensity and functional disability in the VRT group than the other two groups. Especially, VRT exercises activate the function of sensation, which in turn improves the motor function. This principle improves the muscle properties and strengthens the targeted muscles of activation. It is observed that improved muscle function would reduce the pain intensity level and improve functional disability status. VRT commonly works with real-time feedback information to execute and complete the games, which provide a positive environment for the participant to progress to the next level. Overall, this process activates the motor learning of these muscles in a quick level^26,27^. Furthermore, the virtual environment in VRT changes the pain perception level and makes him feel comfortable. The graded improvement in the difficulty level of tasks enhances the attention, concentration, memory, and physical capacity of the participants and leads to improvement in functional status, which were in agreement with some studies^28,29^, but against by Danneels et al.^30^.

Overall, this study observed minimal or no effects on BMP after different types of training protocols and we did not have any evidence for describing the same. The small temporary changes in BMP 2, 4, 6 and 7 are not due to the effects of VRT, SMT, and conventional training protocols. Moreover, Chen CL et al. found that VRT exercises have a positive role in the improvement of bone mineral density and bone mineral content in cerebral palsy children^31^. Furthermore, this study analyzed the impact of virtual reality training on pro-inflammatory biomarkers in PTOA after ACL injury. Our study reports significant improvement in inflammatory biomarkers after VR training. The reports show a decrease in CRP, TNF-α, IL-6, and increase in IL-2 and IL-4 values after this training, these alterations in inflammatory cytokines would be helpful to decrease the inflammatory process in OA, which is in agreement with the study by Yeo et al.^32^.

We have also noticed a mild positive tendency in pain intensity and functional disability in SMT exercise than conventional exercise training. Through the central integration theory, SMT exercises recover the knee muscle strength by stimulating the joint proprioceptors, which in turn improves the joint function and joint stability^33^. Sensory-motor training exercises facilitate the reflex reaction of the knee muscle activities by different phases of exercises. Similar to VR training, SMT exercises and conventional training exercises also not expressed the changes in specific BMPs. Sensory-motor training also shows mild changes in inflammatory biomarkers in PTOA than conventional training. Aguiar et al. noticed that specific SMT exercises modify the pro-inflammatory cytokines, which reduce the inflammatory reaction and lead to decrease pain intensity and improve the joint function in PTOA patients^34^. At the same time, this study observed the positive relation between the pain intensity and inflammatory biomarkers in PTOA subjects, which was in agreement with Imamura et al.^35^.

While executing this study, few limitations were observed such as: first, this study did not include female participants; hence the reports of this study cannot be generalized to the overall population. Secondly, this study didn’t measure the clinical parameters such as range of motion, muscle strength, and Q angle. Third, the association between the clinical findings, bone morphogenic proteins, and inflammatory biomarkers in PTOA after different training protocols has not been analyzed. Finally, the long-term effects, such as after six months of different training protocols, has not been measured.

The reports of our study conclude that training with virtual reality protocol has shown more improvement in pain intensity and functional disability than sensory-motor training in post-traumatic osteoarthritis after the ACL injury. Adding VRT exercises in regular rehabilitation programs shows positive changes in inflammatory biomarkers and mild or zero effect on bone morphogenic proteins. Also, virtual reality training is considered as the latest exercise training technology in the field of sports rehabilitation in different games. Therefore, future studies can be done to investigate the other effects of virtual reality training on different injuries in different games, and also it could be focused on finding the possible mechanisms to improve the BMP in PTOA, which can be justified.

## Data Availability

As per institution policy, the data will not be disclosed.
